# Monitoring Therapy with MEK Inhibitor U0126 in a Novel Wilms Tumor Model in *Wt1* Knockout *Igf2* Transgenic Mice Using ^18^F-FDG PET with Dual-Contrast Enhanced CT and MRI: Early Metabolic Response Without Inhibition of Tumor Growth

**DOI:** 10.1007/s11307-012-0588-5

**Published:** 2012-08-09

**Authors:** Leo G. Flores, Hsin-Hsien Yeh, Suren Soghomonyan, Daniel Young, James Bankson, Qianghua Hu, Mian Alauddin, Vicki Huff, Juri G. Gelovani

**Affiliations:** 1 Department of Experimental Diagnostic Imaging, UT MD Anderson Cancer Center, Houston, TX USA; 2 Department of Imaging Physics, UT MD Anderson Cancer Center, Houston, TX USA; 3 Department of Genetics, UT MD Anderson Cancer Center, Houston, TX USA; 4 1515 Holcombe Blvd, T8.3904, Unit 059, Houston, TX 77054 USA

**Keywords:** Transgenic mice, Wilms tumor, ^18^F-FDG, PET/CT, CT, MRI

## Abstract

**Purpose:**

The understanding of the role of genetic alterations in Wilms tumor development could be greatly advanced using a genetically engineered mouse models that can replicate the development and progression of this disease in human patients and can be monitored using non-invasive structural and molecular imaging optimized for renal tumors.

**Procedures:**

Repetitive dual-contrast computed tomography (CT; intravenous and intraperitoneal contrast), T2-weighted magnetic resonance imaging (MRI), and delayed 2-deoxy-2-[^18^F]fluoro-d-glucose (^18^F-FDG) positron emission tomography (PET) were utilized for characterization of *Igf2* biallelic expression/*Wt1* knockout mouse model of Wilms tumor. For CT imaging, Ioversol 678 mg/ml in 200 μl was administered i.p. followed by 100 μl injected intravenously at 20 and 15 min prior to imaging, respectively. Static PET imaging studies were acquired at 1, 2, and 3 h after i.v. administration of ^18^F-FDG (400 μCi). Coronal and sagittal T1-weighted images (T_E_/T_R_ 8.5/620 ms) were acquired before and immediately after i.v. injection of 0.4 ml/kg gadopentetate dimeglumine followed by T2-weighted images (T_E_/T_R_ 60/300 ms). Tumor tissue samples were characterized by histopathology and immunohistochemistry for Glut1, FASN, Ki67, and CD34. In addition, six Wt1-Igf2 mice were treated with a mitogen-activated protein kinase (MEK) inhibitor U0126 (50 μmol/kg i.p.) every 4 days for 6 weeks. ^18^F-FDG PET/CT imaging was repeated at different days after initiation of therapy with U0126. The percent change of initial tumor volume and SUV was compared to non-treated historic control animals.

**Results:**

Overall, the best tumor-to-adjacent kidney contrast as well as soft tissue contrast for other abdominal organs was achieved using T2-weighted MRI. Delayed ^18^F-FDG PET (3-h post ^18^F-FDG administration) and dual-contrast CT (intravenous and intraperitoneal contrast) provided a more accurate anatomic and metabolic characterization of Wilms tumors in Wt1-Igf2 mice during early development and progression of renal tumors. Over the 8-month period, 46 Wt1-Igf2 mice and 8 littermate control mice were studied. Renal tumors were identified in 54.3 % of Wt1-Igf2 mice between post-natal 50–100 days. In 35.6 % of Wt1-Igf2 mice, tumors were localized in the right kidney; in 24 %, in the left kidney, while 40.4 % of Wt1-Igf2 mice had bilateral kidney tumors. Metastatic lesions were identified in 15.4 % of Wt1-Igf2 mice. Increased levels of Glut1 and IGF1R expression, high Ki67 labeling index, and a dense network of CD34+ microvessels in renal tumors was consistent with increased ^18^F-FDG accumulation. Treatment with a MEK 1/2 inhibitor U0126 did not cause the inhibition of tumor growth as compared to untreated animals. However, after the first three to four doses (~2 weeks of treatment), a decrease in ^18^F-FDG SUV was observed, as compared to pre-treatment levels (*p* < 0.05, paired Student *t* test), which constitutes a metabolic response. Six weeks later, despite continuing therapy, the ^18^F-FDG SUV increased again to previous levels.

**Conclusions:**

The optimized dual contrast PET/CT imaging with early post i.v. and i.p. contrast CT and 3 h delayed PET imaging after ^18^F-FDG administration provides a sensitive and reliable method for detecting early tumor lesions in this endogenous mouse model of Wilms tumor and for monitoring their growth in response to targeted therapies. Therapy with MEK inhibitor U0126 produces only a transient inhibition of tumor glycolytic activity but does not inhibit tumor growth, which is due to continuing IGF2-induced signaling from IGF1R through the PI3K-AKT-mTOR pathway.

## Introduction

Wilms tumor or nephroblastoma, an embryonal neoplasm of the kidney is the second most common intra-abdominal cancer in children and accounts for more than 95 % of all tumors of the kidney in the pediatric patients [1]. The pathogenesis of Wilms tumor is complex yet provides insight into the relationship of embryology and oncogenesis. The understanding of the role of genetic alterations in tumor development has advanced slowly due to the need of a suitable Wilms tumor mouse model. Recent technological advances now allow manipulation of the mouse genome to constitutively or conditionally alter the expression of crucial genes leading to development of a particular tumor model. The known mutations involved in Wilms tumor are inactivation of *WT1* due to germline and/or somatic mutation, somatic stabilizing CTNN1B mutations, somatic deletion of WTX, and somatic p53 mutation [2].

Subcutaneous (s.c.) tumor xenograft models of Wilms tumor using SK-NEP-1 and G401 cell lines have been employed extensively to assess the effectiveness of new drugs and various treatment approaches [3–9]. These s.c. Wilms tumor models result in highly reproducible data because tumor growth can be visually monitored and easily measured. However, s.c. tumor xenograft models do not adequately replicate natural organotypic tumor stromal microenvironment achieved by orthotopic xenograft models of Wilms tumor [10], which, however, are not always suitable to studies of the mechanisms of oncogenesis, tumor maintenance, progression, and response to therapy [11, 12]. Furthermore, recent studies demonstrated that the SK-NEP-1 cell line, previously thought to represent anaplastic Wilms tumor, is instead related to Ewing sarcoma [8] and that the G401 cell line is actually a rhabdoid kidney tumor [9]. Thus, the availability of adequate orthotopic xenograft models of Wilms tumor is very limited.

In contrast, transgenic and knockout tumor models enable studies on organ-specific oncogenesis, provide information on how an isolated genetic alteration contributes to mechanisms of malignant transformation and progression, and lay the ground work for targeted therapies. However, due to the lack of visual control and easy access for caliper-based measurements, monitoring of tumor growth in both orthotopic and, especially, endogenous tumor models requires repetitive non-invasive anatomic and/or functional imaging [13].

Usually, for longitudinal characterization of orthotopic and endogenous mouse models of renal tumors, mice are euthanized at different time points after the initial implantation of tumor cells or at different weeks after birth (in the case of endogenous tumors) and kidneys are harvested for tumor detection, measurements, and histopathology [14, 15]. Studies in orthotopic Wilms tumor models in mice relied on palpation for monitoring tumor growth, which allowed for detection of only large tumors (approximately 3 cm^2^, 5 weeks after cell injection) [3]. High frequency ultrasound imaging (USI) allowed detection and morphologic characterization of orthotopic Wilms tumor xenografts as less than 2 mm^2^ at 3–5 weeks after tumor cell implantation [16], but the reproducibility of USI is low because it is operator-dependent. Orthotopic xenografts of human renal carcinoma cells transduced with *firefly luciferase* (FLuc) reporter gene allowed for reliable, rapid, and noninvasive longitudinal monitoring of tumor growth *in vivo* with bioluminescence imaging (BLI) [17]. Because transgenic mouse models of renal carcinomas expressing reporter genes for BLI have not been developed yet, other approaches using non-invasive anatomic and/or molecular imaging should to be evaluated for tumor detection and therapy monitoring. Furthermore, BLI lacks the depth information and is not suitable for determination of tumor size based on BLI signals.

Here, we describe an optimized imaging methodology with dual contrast computed tomography (CT; intravenous and intraperitoneal), T2-weighted magnetic resonance imaging (MRI), and delayed 2-deoxy-2-[^18^F]fluoro-d-glucose (^18^F-FDG) positron emission tomography (PET) for characterization of a recently developed *Igf2* biallelic expression/*Wt1* knockout mouse model of Wilms tumor [18], which allowed for more accurate repetitive anatomic and metabolic imaging during the early development and progression of Wilms tumors and their responses to experimental targeted therapies. We demonstrate that *Wt1* ablation and *Igf2* upregulation in tumors results in up-regulation of glucose utilization during initial stages of tumor development, followed by a gradual decrease in tumor glycolytic activity, consistent with the development of large areas of hemorrhagic necrosis. Furthermore, we demonstrate that therapy with a mitogen-activated protein kinase (MEK) inhibitor U0126 causes a transient decrease in tumor glycolytic activity, consistent with downregulation of signal transduction downstream MEK, but does not significantly affect the rate of tumor growth.

## Materials and Methods

### Tumor Model

All studies involving animals were performed in accordance with regulations of the Institutional Animal Care and Use Committee (IACUC) at the UT MD Anderson Cancer Center. The new endogenous Wilms tumor model was recently described [18]. Briefly, tumor-watch cohort (*Wt1* ^*–/fl*^ *H19* ^*+/–m*^ *Cre-ER* ^*TM*^) and littermate controls (*Wt1* ^*+/fl*^ *H19* ^*+/–m*^ *Cre-ER* ^*TM*^, *Wt1* ^*–/fl*^ *H19* ^*+/–m*^, and *Wt1* ^*+/fl*^ *H19* ^*+/–m*^) were generated by crossing *Wt1* ^*fl/fl*^ *H19* ^*–/–*^ females with *Wt1* ^*+/–*^ *Cre-ER* ^*TM*^ males. The *Wt1* ^*fl*^ allele, upon induction of Cre-recombinase activity by tamoxifen, is recombined, resulting in an inactive protein. The H19^–^ mouse strain was provided by S. Tilghman (Princeton University, Princeton, New Jersey, USA) and carries a deletion in the imprinting control region, ICR1, for Igf2, resulting in loss of imprinting. Maternal inheritance of this deletion results in expression from the normally silenced maternal allele and biallelic expression of *Igf2* [19]. Cohorts of mutant and littermate controls were treated with 1 mg/40 g TM at E11.5. The offspring were termed “Wt1-Igf2 mice” and were monitored for tumor development as described further. The mice were housed at an AAALAC-accredited small animal vivarium facility in isosexual groups of 2 to 5, receiving regular chow and water *ad libitum*; the room was kept on a 12:12-h light/dark cycle and temperature was maintained at 22 °C with relative humidity between 30 % and 70 %.

### PET/CT Imaging Protocol

PET/CT imaging was performed using Inveon small animal PET/CT instrument (Siemens, Knoxville, TN). The Wt1-Igf2 mice were anesthetized with isoflurane (2 % in 98 % oxygen) and their temperature kept at 38 °C with a heating pad using T/PUMP and T/PADS (Gaymar Industries, Inc., Orchard Park, NY).

An iodine-based contrast agent Ioversol (678 mg/ml; Mallinckrodt, Hazelwood, MO) 200 μl was administered intraperitoneally (i.p.) followed by 100 μl injected intravenously at 20 and 15 min prior to imaging, respectively. The microCT imaging parameters were: X-ray voltage of 80 kVp, anode current of 500 μA, and exposure time of 300–350 milliseconds of each of the 360 rotational steps. CT images were reconstructed using Shepp–Logan algorithm. The volume of individual tumor lesions was measured by drawing regions of interest (ROI) outlining individual tumor lesions on each CT slice, summing the areas of individual ROI measured in all slices and multiplying by slice thickness to calculate the lesion volume. Similar window and center settings were used for determination of tumor boundaries to minimize errors associated with volumetric analysis of images obtained at different days of the study in different animals.

Static PET imaging studies were acquired for 15 min at 1, 2, and 3 h after i.v. administration of ^18^F-FDG (400 μCi in 100 μl of saline) and reconstructed using two-dimensional ordered subsets expectation maximization (2DOSEM) algorithm with four iterations and 16 subsets. PET/CT image fusion and analysis were performed using vendor software Inveon Research Workplace version 3.0 (Siemens, Knoxville, TN). Tumor standardized uptake value (SUV) for ^18^F-FDG was calculated by drawing ROI outlining individual tumor lesions on CT images and transposing them to corresponding PET images, as previously described [20].

### MR Imaging Protocols

The Wt1-Igf2 mice were anesthetized with 1 % to 3 % isoflurane and placed prone and head first on the positioning sled. Electrocardiography leads were positioned on the forepaws and tail, and respiratory bellows were positioned over the abdomen. Imaging was done using 4.7-T small animal MRI scanner (Biospec, Bruker Biospin MRI, Billerica, MA) with standard gradient (60 mm ID) volume RF recoil (35 mm ID) configurations. Coronal and sagittal T1-weighted images (T_E_/T_R_ 8.5/620 ms) were acquired before and immediately after injection via tail vein catheter of 0.4 ml/kg gadopentetate dimeglumine (Magnevist; Berlex Laboratories, Inc., Montville NJ, USA); thereafter T2-weighted images (T_E_/T_R_ 60/300 ms) were acquired. Multi-slice acquisition was performed with a field-of-view of 50 × 37.5 mm and a matrix of 256 × 192 points, slice thickness of 1 and 0.25 mm gap between slices. All image analyses were performed using ImageJ software (http://rsb.infp.nih.gov/ij).

### Histopathology and Immunohistochemistry

Moribund mice were humanely sacrificed and after gross pathological examination tumor tissues sampled, fixed in 2 % paraformaldehyde and embedded in paraffin. Five-micrometer-thick tumor tissue sections were obtained using the RM2255 microtome (Leica, Germany), deparafinized, and step-wise rehydrated in PBS. The sections were blocked with either goat or hose serum for 2 h at room temperature followed by incubation overnight at 4 °C with primary rabbit monoclonal antibodies to Glut 1 (H-43) or fatty acid synthase (C20G5; all from Santa Cruz, CA), followed by secondary biotinylated goat anti-rabbit antibodies (BA-1000; Vector Laboratories, CA). Incubation with mouse monoclonal antibodies to Ki67 (MM1) was followed by biotinylated horse anti-mouse antibodies (BA-2001; both from Vector Laboratories, CA). Goat antibodies to CD34 (AF4117; R&D Systems, CA) and secondary rabbit anti-goat biotinylated antibodies (BA-5000 Vector Laboratories, CA) were used to detect microvasculature. Avidin-peroxidase and 3′3-diaminobezidine were from Vectastain Elite kit (Vector Laboratories, CA). Hematoxylin was used as a counterstain.

### Monitoring Treatment Response

After detection of the developing tumors using ^18^F-FDG PET/CT imaging, the Wt1-Igf2 mice (*N* = 6) were treated with a mitogen-activated protein kinase (MEK) inhibitor U0126 (50 μmol/kg i.p. in 200 μl of 40 % DMSO in saline) every 4 days for 6 weeks [21]. ^18^F-FDG PET/CT imaging was repeated at different days after initiation of therapy with U0126. The percent change of initial tumor volume and SUV changes were calculated, plotted over time, and compared to non-treated animals.

## Results

### Optimization of CT and MRI Imaging Protocols

**Fig. 1:**
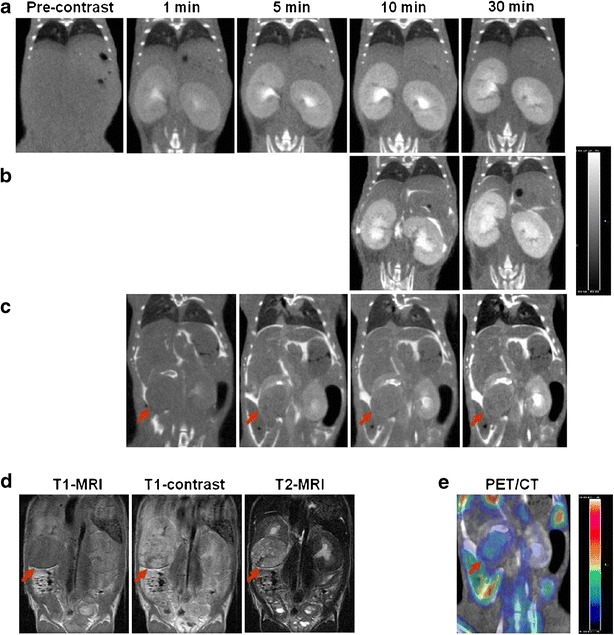
*In vivo* MicroCT, MRI, and PET/CT in C57BL/6 and Wilms tumor model. Coronal CT images obtained **a** before and 1, 5, 10, and 30 min after i.v. injection of contrast in a normal C57BL/6 mouse; **b** at 10 and 30 min after i.v. and i.p. injection of contrast in a normal C57BL/6 mouse; **c** obtained 1, 5, 10, and 30 min after i.p. and i.v. dual-contrast administration in a Wt1-Igf2 mouse. **d** T1-weighted MR images before and after administration of an intravenous contrast (Gd-DTPA) and T2 image obtained in the same Wt1-Igf2 mouse (shown in **c**) and **e** the corresponding image of ^18^F-FDG microPET/CT with dual contrast. The CT images are shown in *gray scale* with the same window and center. *Arrows* point at the renal tumor mass.

Initial CT imaging studies were conducted in normal C57BL/6 mice (*N* = 6) to optimize the timing and volume of i.v. injection of contrast agent. Pre-contrast CT images resulted in a very poor soft tissue contrast (Fig. 1a). After administration of i.v. contrast, increasing attenuation signal from the renal cortico-medullary tissue was observed, reaching a plateau after about 10 min, which is consistent with the excretory phase of a nephrographic study [22]. However, the identification of margins of organs adjacent to kidneys using only the i.v. contrast was still not feasible. Intraperitoneal co-administration of contrast prior to i.v. contrast administration resulted in a significant improvement in delineation of margins of kidneys and other abdominal organs (Fig. 1b). CT images obtained 30 min after combined i.p. and i.v. dual-contrast administration provided a fairly good delineation of renal tumor masses, which presented as characteristically hypodense areas with clearly identifiable borders with contrast-enhancing functional renal parenchyma (Fig. 1c).

In Wt1-Igf2 mice, the renal tumor signal intensity on non-enhanced T1 MR images was homogenous and only slightly lower than that of the adjacent cortico-medullary renal tissue, which did not provide sufficient tumor-to-adjacent kidney contrast (Fig. 1d). In T1-weighted contrast-enhanced MR images, both tumor and adjacent kidney signal intensities were similarly increased, which provided almost no contrast between the tumor and peritumoral kidney tissue. However, the T1 signal heterogeneity in the tumor tissue was sometimes suggestive of tumor margins.

Overall, the best tumor-to-adjacent kidney contrast as well as soft tissue contrast for other abdominal organs was achieved using T2-weighted MRI (Fig. 1d). On T2-weighted MR images, tumor tissue had an increased signal intensity, which contrasted with relatively low T2 signal intensity of the adjacent renal cortico-medullary tissue. A clearly distinguishable tumor margin was usually observed on T2-weighted MR images.

### Optimization of ^18^F-FDG PET/CT Imaging Protocol

**Fig. 2:**
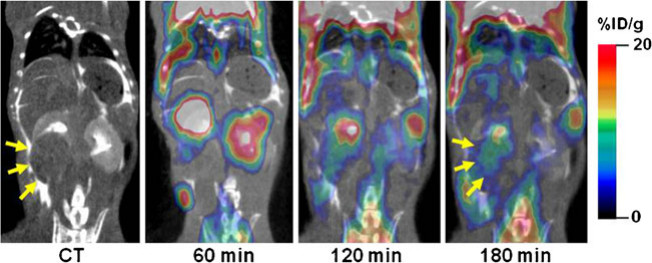
*In vivo* dual-contrast microCT and microPET/CT of a Wt1-Igf2 mouse with a renal (Wilms) tumor acquired at 60, 120, and 180 min after i.v. administration of ^18^F-FDG. *Arrows* point at the renal tumor mass.

In a preliminary PET study involving 4 Wt1-Igf2 mice, delayed acquisition of PET images up to 3 h post ^18^F-FDG administration resulted in a significant clearance of ^18^F-FDG from the kidneys and “unmasking” of areas or radioactivity accumulation in the tumor tissue (Fig. 2). ^18^F-FDG PET/CT imaging of Wt1-Igf2 mice using delayed image acquisition protocol provided further characterization of tumor glucolytic activity (Fig. 1e).

### Immunohistochemical Characterization of the Tumor Model

**Fig. 3:**
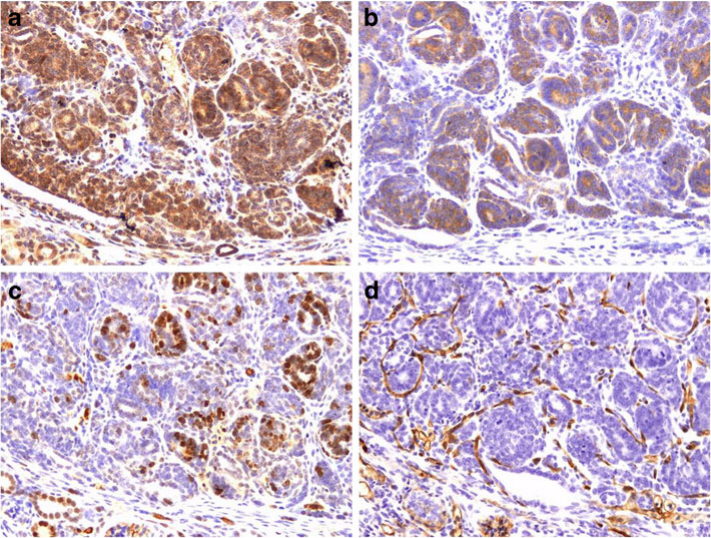
Immunohistochemical characterization of Wilms tumor tissue for expression of **a** Glut 1, **b** fatty acid synthetase (FASN), **c** Ki67 (proliferating cells), and **d** CD34^+^ microvasculature.

The level of Glut1 expression in renal tumors of Wt1-Igf2 mice was uniformly increased in the tumor cells (Fig. 3a), which contributes to the mechanism of ^18^F-FDG accumulation. The level of fatty acid synthase (FASN) expression in tumor cells was only moderately increased in the majority of tumor cells (Fig. 3b), which suggests that PET/CT imaging with radiolabeled acetate or choline may not be effective, but should also be explored. Proliferative activity in these endogenous Wilms tumors was heterogeneous; higher density of Ki67 labeling was typically observed in the periphery of individual tumor lesions (Fig. 3c), which suggests that PET/CT imaging with ^18^F-FLT and other radiolabeled nucleoside analogues should be explored in the future. Significant neoangiogenesis was observed in tumors, as evidenced by a dense network of CD34^+^ microvasculature (Fig. 3d), which should contribute to contrast enhancement on CT; however, CT contrast enhancement of these tumors was significantly less than in normal kidney parenchyma.

### Long-Term Monitoring of Tumor Development by PET/CT with ^18^F-FDG

**Fig. 4:**
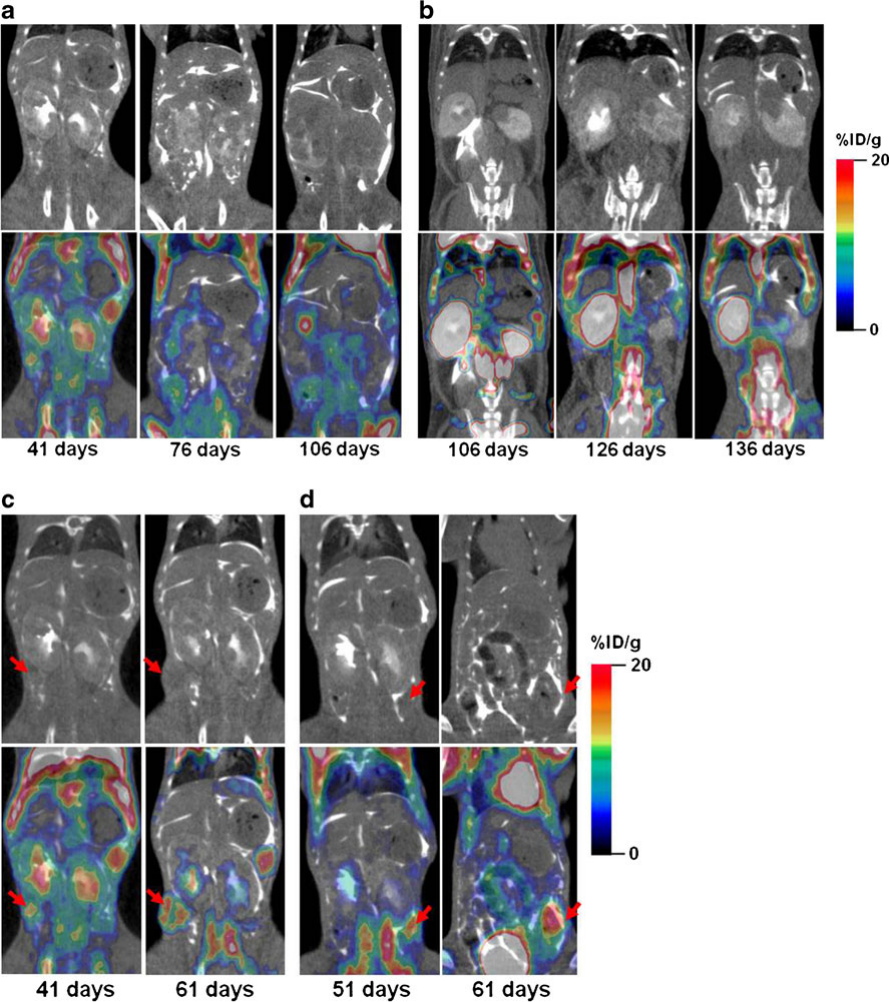
Repetitive microPET/CT with ^18^F-FDG and dual-enhanced CT of bilateral renal tumors in Wt1-Igf2 mice. **a** CT images obtained at 41 days demonstrate a significant deformation of renal pelvis and collicular architecture of both kidneys due to presence of multi-focal tumor lesions with increased glycolytic activity on PET. PET/CT Images of the same animal obtained at 76 and 106 days demonstrate a significant increase in size of individual tumor lesions, coalescence of tumor lesions into larger tumor masses with apparent decrease in glycolytic activity in most tumors. **b** In some animals, PET/CT images revealed a significant decrease in renal clearance of ^18^F-FDG, which manifested by high persistent radioactivity in the obstructed kidney. **c** PET/CT images of a Wt1-Igf2 mouse with extrarenal glycolytically active metastatic tumor lesion growing intra-abdominally on the right side at 41 days; this tumor lesion has grown through the abdominal wall into the subcutaneous space and remained glycolytically active 20 days later. **d** PET/CT images of another Wt1-Igf2 mouse with glycolytically active extrarenal metastasis growing intra-abdominally on the left side at 51 days, and invading the abdominal wall muscles 10 days later.

Over the 8-month period, 46 Wt1-Igf2 mice and 8 littermate control mice were studied. Amongst the Wt1-Igf2 mice, 48 % was imaged once and 52 % was imaged at least three times per month using ^18^F-FDG PET/CT with dual (i.p. and i.v.) contrast enhanced CT (Fig. 4a). Renal tumors were identified in 54.3 % of Wt1-Igf2 mice between post-natal 50–100 days. Renal tumors were multifocal in 65.4 % of Wt1-Igf2 mice, while 34.6 % had a single detectable lesion only in one kidney. In 35.6 % of Wt1-Igf2 mice, tumors were localized in the right kidney; in 24 %, in the left kidney, while 40.4 % of Wt1-Igf2 mice had bilateral kidney tumors. In Wt1-Igf2 mice, the incidence of tumors was slightly higher in males (56 %) than females (44 %). Invariably, the Wt1-Igf2 mice developed hydronephrosis due to tumor-induced obstruction of urine outflow from the renal pelvis or ureters. This caused a significant retention of ^18^F-FDG-derived radioactivity in the kidney pelvis and parenchyma, which often confounded the interpretation of PET/CT images (Fig. 4b). Extrarenal tumors were identified in 15.4 % of Wt1-Igf2 mice. The typical localization of extrarenal intra-abdominal tumors was in the lower abdominal peritoneal recesses, which may be due to local dissemination of tumor cells developing implant metastases (Fig. 4c, d).

**Fig. 5:**
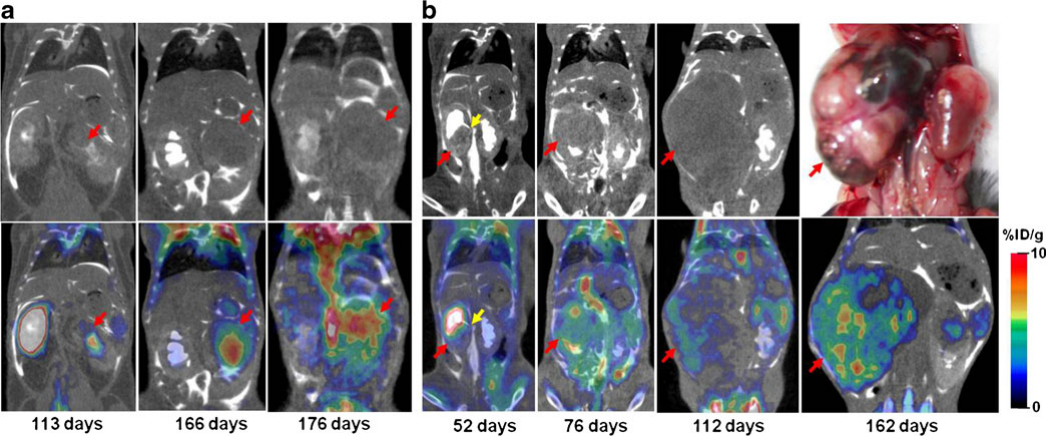
MicroPET with double enhanced CT of Wt1-Igf2 mice with bilateral renal tumors obtained at different times during development. **a** In one Wt1-Igf2 mouse obstractive hydronephrosis was detectable in the right kidney, which manifested by retention of ^18^F-FDG and CT contrast in the renal pelvis; in the left kidney, the developing tumor has gradually destroyed the whole kidney and retained a glucolytically active core. **b** In another Wt1-Igf2 mouse a large tumor that completely destroyed the right kidney had multiple hemorrhagic cysts (evident at gross pathologic examination), which explains the relatively lower average glucolytic activity.

A significant impairment of renal excretory function of the affected kidney(s) was observed in all animals at later stages of tumor development, as evidenced by the absence of contrast-enhancement of cortico-medullary renal tissue and by a significant decrease or absence of contrast material outflow from the affected kidney. Further tumor growth resulted in an almost complete destruction of kidney anatomy and the formation of large heterogeneous tumor masses with multiple urine-filled hemorrhagic cysts (Fig. 5)**.**

**Fig. 6:**
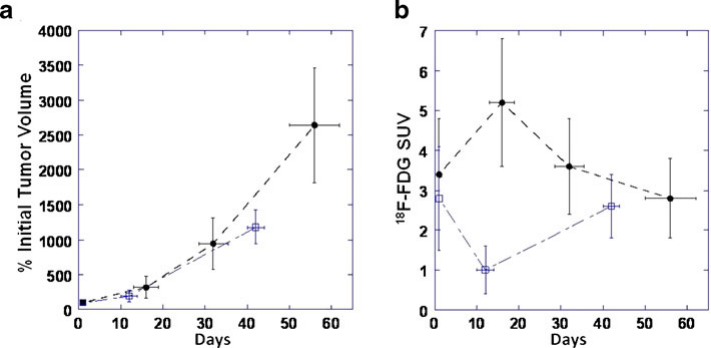
Quantification of **a** tumor growth expressed as % initial tumor volume and **b** ^18^F-FDG SUV over time (days post initial initiation of therapy) in Wt1-Igf2 mice treated with MEK inhibitor UO126 (*open squares*) and control littermate mice receiving saline (*filled circles*).

To facilitate the comparison of tumor growth trends between different animals, tumor volumes measured in individual animals at different days after initial tumor detection were normalized to the initial tumor volume measured at the time of detection and expressed as % tumor volume at the time of initial detection (which was set to 100 %). Also, to facilitate the comparison of ^18^F-FDG SUV trends, the average tumor ^18^F-FDG SUVs was measured in individual animals at different days after initial tumor detection (Fig. 6a). Comparison of growth and glycolytic activity of Wilms tumors in these transgenic mice over time revealed that tumors exhibited a trend for increasing ^18^F-FDG SUV during the first 2 weeks after the initial detection (Fig. 6a), when tumor volume was small (3–4 mm in diameter). However, during the following 2 weeks of monitoring, the average tumor ^18^F-FDG SUV significantly decreased, which was consistent with the development of necrotic and cystic tumor regions and moderate-to-high glycolytic activity persisting within viable tumors regions. During the subsequent 4 weeks of monitoring, the average tumor ^18^F-FDG SUV gradually increased, albeit not as dramatically as during the initial phase of tumor growth.

### Monitoring Therapy with a MEK Inhibitor

For the assessment of treatment response, we relied on morphometry and ^18^F-FDG SUV of the most prominent tumor lesions. Treatment with a MEK 1/2 inhibitor U0126 did not cause the inhibition of tumor growth, as compared to untreated animals. However, after the first three to four doses (~2 weeks of treatment), a decrease in ^18^F-FDG SUV was observed as compared to pre-treatment levels (*p* < 0.05, paired Student *t* test), which constitutes a metabolic response. Six weeks later, despite continuing therapy, the ^18^F-FDG SUV increased again to previous levels (Fig. 6b).

## Discussion

In this study, combined anatomical and molecular imaging was utilized and optimized for characterization of a new endogenous mouse model of Wilms tumor. In this tumor model, somatic inactivation of the *Wt1* gene occurs in the context of bi-allelic over-expression of the *Igf2* gene in a small subset of differentiating metanephric mesenchymal cells. This is achieved by a conditional ablation of the *Wt1* gene via dose-dependent, tamoxifen-inducible, Cre-mediated recombination of the *Wt1* ^*fl*^ allele, which results in a *Wt1Δ* allele encoding a truncated mutant WT1 protein that is functionally indistinguishable from the *Wt1* ^*–*^ allele [18, 23]. Such an approach allows for normal embryonal development of functional kidneys, which warrants postnatally viable offspring and mimics the alterations observed in human tumors. Previous studies demonstrated that Wt1-Igf2 tumors recapitulate the histology and signature of gene expression of human Wilms tumors [18]. Because combined alterations in IGF2 and WT1 genes causes dysregulation of ERK signaling pathway, similar to that observed in human tumors, the current model can be used for the assessment of therapeutic efficacy of drugs targeting IGF and ERK signaling pathways.

However, the variability of onset and dynamics of Wilms tumor development in these Wt1-Igf2 mice dictates the need for highly sensitive non-invasive imaging, to enable early detection of tumor lesions in kidneys and for longitudinal monitoring of their progression or regression for the assessment of novel therapeutic strategies targeting IGF and ERK signaling pathways. Detection of orthotopic and endogenous Wilms tumors using contrast-enhanced CT or T1-weighted MRI is challenging because kidneys are involved in clearance of CT and MRI contrast agents. Delineation of renal tumor masses on contrast-enhanced CT and MR images is based on a negative contrast, which is due to higher perfusion and excretion of contrast agent by the normal kidney tissue, as compared to tumor tissue lacking the excretory function [24]. Therefore, renal tumors are detectable more reliably using T2-weighted MRI, which is due to significantly higher signal intensity of tumor tissue, as compared to normal kidney tissue [25]. The results of current imaging studies in Wt1-Igf2 mice have confirmed the higher accuracy of T2-weighted MRI for early detection, delineation of margins, and for assessment of structural heterogeneity of Wilms tumors, as compared to T1-weghted MRI and CT with or without i.v. contrast enhancement.

Previously, Johnson et al. [26] reported that administration of intraperitoneal contrast results in an opacification of intraperitoneal fluid and a significant improvement of visualization of boundaries of different abdominal organs. The current study also demonstrated the efficacy of dual contrast-enhanced CT (using co-administration i.v. and i.p. contrast) for detection and monitoring of renal tumors in Wt1-Igf2 mice. Dual-contrast CT provided a better delineation of tumor boundaries in relation to neighboring organs, especially in advanced Wilms tumors causing significant displacement of abdominal organs. Furthermore, as an alternative to T2-weighted MRI, dual-contrast enhanced CT was implemented as part of a PET/CT imaging protocol, which simplified handling of the experimental animals between different imaging modalities and minimized errors of inter-modality co-registration of anatomical and functional (molecular) images.

Small animal PET and PET/CT imaging with different radiotracers have been widely used for detection, characterization of metabolic and signaling abnormalities, and for monitoring of various therapeutic approaches in transgenic mouse tumor models [27–33]. In particular, PET imaging with 2′-[^18^F]-fluoro-2′-deoxy-d-glucose (^18^F-FDG) has been extensively utilized for detection of tumors with increased glycolytic activity, which results from de-regulated oncogenic signaling [34], which causes the “Warburg” effect [35]. Application of ^18^F-FDG PET for early detection and characterization of kidney tumors in mice is challenging, due to predominantly renal clearance of ^18^F-FDG from the circulation, which generates high level of normal kidney radioactivity and obscures small renal tumor masses. Therefore, in orthotopic renal carcinoma models ^18^F-FDG PET has been used mainly to detect distal metastatic lesions [36]. However, PET/CT imaging provides significantly more accurate diagnostic information, as compared to CT or PET imaging alone. In clinical setting, PET/CT has been useful in identifying the most metabolically active portions of tumors for targeting of biopsies, staging, monitoring responses to chemotherapy, and for disease surveillance [37, 38]. Also, previous clinical studies demonstrated that administration of CT contrast agent does not significantly impair the quality of ^18^F-FDG PET images [39].

The current study demonstrated the applicability of ^18^F-FDG PET/CT for detection of early stage developing WT lesions as well as limitations for monitoring their growth in this endogenous mouse model of Wilms tumor. The rationale for using PET/CT imaging with ^18^F-FDG in this tumor model was based on the overexpression of *Igf2* and deletion of *Wt1*. Mutational inactivation of WT1 tumor suppressor also leads to activation of the IGF-1R signaling and its downstream targets such as Grb2 which signals through Ras, Raf, and ultimately mitogen-activated protein kinase (MAPK), which is involved in cellular events such as growth, differentiation, and stress response [40]. Growth factors such as IGF1 and IGF2 drive HIF-1α through MEK1 and MEK2 signaling, that influences tumor glucolytic activity [41]. Furthermore, MAPK signaling facilitates malignant transformation, drives growth and progression of tumor and enhances its ability to invade and metastasize [42, 43]. This explains, at least in part, why several Wt1-Igf2 mice also developed not only renal tumors, but also extrarenal metastatic tumor lesions. These metastatic tumors may have initially developed by peritoneal dissemination of tumor cells invading through the renal capsule or released as the result of rupture of subcapsular cystic tumor lesions. However, at later stages of growth, these metastatic lesions have grown through the abdominal muscles and often extended into the subcutaneous space, which demonstrates their invasive phenotype.

The upregulation of *Igf2* in this Wilms tumor model causes increased signaling through the IGF-IR via pIRS1 and pERK1/2, which drives the proliferation of these abnormal cells [18]. It is noteworthy, that a significant fraction of human WTs also exhibits increased pIRS1 and pERK1/2 [18]. ERK is a downstream component of a signaling cascade that is activated by the Raf serine/threonine kinases. Raf activates the MAPK/ERK kinase (MEK)1/2 dual-specificity protein kinases, which then activate ERK1/2 [44]. Therefore, in this study we used U0126, a highly selective inhibitor of MEK1 and MEK2 [21], to assess its therapeutic efficacy in this Wilms tumor model and to investigate whether the U0126 would induce changes in tumor glycolytic activity that can be used as a potential pharmacodynamic biomarker. Using repetitive ^18^F-FDG PET/CT with dual contrast CT, we observed only transient inhibition of glucolytic activity during the first week of therapy with U0126 at doses that have been proven effective in therapy of other tumor types [21]. However, no significant differences in the dynamics of tumor growth were observed between U0126 treated and untreated animals. The lack of therapeutic efficacy of U0126 in this Wilms tumor model and only a transient decrease of glucolytic activity could be explained, at least in part, by continuing IGF2-induced signaling from IGF1R through the PI3K-AKT-mTOR pathway [45], bypassing the block in MAPK signaling. Although, our previous studies in this Wilms tumor model demonstrated no significant upregulation of Akt phosphorylation [18], it is possible that persistent PI3K-AKT-mTOR signaling may compensate for the inhibited MAPK signaling during therapy with a MEK inhibitor. The current study demonstrated that genetic and signaling aberrations in this Wilms tumor model ultimately lead to upregulation of glucose transporter (Glut1) expression and hexokinase activity, which explains increased ^18^F-FDG accumulation and enables the detection of early stage tumor lesions. However, at later stages of tumor development, the observed variability of ^18^F-FDG accumulation in tumors reflects the heterogeneity of GLUT1 expression in a polycystic-hemorrhagic tumor tissue. Our findings are in agreement with previous studies in Wilms tumor patients, demonstrating that ^18^F-FDG PET was effective in detection of both primary tumors, as well as residual disease after therapy and disease extent at relapse [46, 47].

Because this Wilms tumor model is characterized with a moderately increased tumor Ki67 labeling index, PET/CT imaging with ^18^F-FLT may be suitable for monitoring tumor proliferative activity. Also, the level of FASN expression in this model of Wilms tumor was only moderately increased, which suggests that PET/CT imaging with radiolabeled acetate and choline may not be suitable for detection and therapy monitoring in this Wilms tumor model. To date, there are no published reports on the efficacy of radiolabeled acetate and choline for PET/CT imaging of Wilms tumors in human subjects. As PET/MRI hybrid imaging systems became more widely available for pre-clinical and clinical applications [48, 49], T2-weighted MRI and PET using metabolic radiotracers such as ^18^F-FDG or alternative radiotracers with significantly less renal clearance (i.e., ^11^C-acetate) should further be explored for detection and longitudinal monitoring of treatment responses of Wilms tumors.

In summary, renal tumors developing in Wt1-Igf2 mice carry the same alterations that occur in human tumors, thus recapitulating the process of development and progression of Wilms tumor in human patients and providing a highly relevant model for testing new molecular targeted therapies. Renal tumors in Wt1-Igf2 mice accumulate ^18^F-FDG due to upregulation of Glut-1 and hexokinase activity. The optimized PET/CT imaging with dual-contrast CT and delayed acquisition of PET images at 3 h post ^18^F-FDG administration provides a sensitive and reliable method for detecting early kidney tumors in this Wilms tumor model. Therapy with MEK inhibitor U0126 produces only a transient inhibition of tumor glycolytic activity but does not inhibit tumor growth, which is most likely due to continuing IGF2-induced signaling from IGF1R through the PI3K-AKT-mTOR pathway.
